# Long-Term Sequelae of Frostbite—A Scoping Review

**DOI:** 10.3390/ijerph18189655

**Published:** 2021-09-14

**Authors:** Ivo B. Regli, Giacomo Strapazzon, Marika Falla, Rosmarie Oberhammer, Hermann Brugger

**Affiliations:** 1 Institute of Mountain Emergency Medicine, Eurac Research, 39100 Bolzano, Italy; Giacomo.strapazzon@eurac.edu (G.S.); marika.falla@unitn.it (M.F.); Hermann.brugger@eurac.edu (H.B.); 2 Department of Anaesthesia and Intensive Care, “F. Tappeiner” Hospital, 39012 Merano, Italy; 3 Department of Anaesthesia and Intensive Care, Medical University Innsbruck, 6020 Innsbruck, Austria; 4 Center for Mind/Brain Sciences, University of Trento, 38123 Rovereto, Italy; 5 Department of Anaesthesia and Intensive Care, Hospital of Brunico, 39031 Brunico, Italy; rosmarie.oberhammer@sabes.it

**Keywords:** frostbite, cold injury, pathophysiology, neuropathic pain, nociceptive pain, arthritis, vasospasm

## Abstract

Frostbite is tissue damage caused by freezing temperatures and constitutes an important cause of morbidity in cold climate zones and high altitude. The direct effects of sub-zero temperatures lead to tissue freezing, electrolyte shifts and pH alterations, microvascular damage, and eventually to cell death. Upon rewarming, inflammatory reperfusion injury and thrombosis may lead to further tissue damage. Several studies and various case reports show that many patients suffer from long-term sequelae such as vasomotor disturbances (associated with susceptibility to refreezing), and neuropathic and nociceptive pain, as well as damage to skeletal structures. There are still many uncertainties regarding the pathophysiology of these sequelae. It has been shown that the transient receptor potential channel (TRP) family plays a role in cold allodynia. Botulinum Toxin type A (BTX-A) injections have been reported to be beneficial in vasomotor and neuropathic disturbances secondary to frostbite. Epidural sympathetic block has been used for short-term treatment of frostbite induced chronic pain. Furthermore, amitriptyline, gabapentinoids, and duloxetine may have some benefits. Frostbite arthritis clinically resembles regular osteoarthritis. In children there is a risk of epiphyseal cartilage damage leading to bone deformities. Despite some promising therapeutic concepts, the scarcity of data on frostbite long-term sequelae in the literature indicates the need of more in-depth studies of this pathology in all its aspects.

## 1. Background

Frostbite is defined as tissue-freezing caused by heat loss sufficient to cause ice-crystal formation in superficial or deep tissue [1]. The severity of frostbite injury depends on environmental temperature, wind chill factor, and length of exposure [2]. Due to their exposed and peripheral anatomical position, frostbite mainly affects distal upper and lower extremities, ears, nose, and cheeks [3]. There is a wide spectrum of clinical presentation of frostbites, ranging from injuries that resolve completely without any consequences, to injuries that result in major limb amputation [4]. However, even without significant tissue loss, patients may suffer from long-term sequelae after having suffered a frostbite injury. These can include vasomotor disorders, neuropathic and nociceptive pain, and frostbite arthritis [5]. Thus far, long-term sequelae of frostbite have been little explored.

This review aims to elucidate the rather neglected aspect of long-term sequelae of frostbite—its epidemiology, its pathophysiology, and its treatment.

### 1.1. Epidemiology

There are no data on the overall incidence of frostbite. Thus far, the oldest recorded frostbite injury was found in the little toe of a 5300 years-old mummified body found on a glacier in the Italian Alps [6]. In military medicine, frostbite has been a significant factor of morbidity since ancient times, e.g., during the crossing of the Alps of Hannibal’s soldiers [4]. In the early 19th century, Dr. Dominique Jean Larrey, Napoleon Bonaparte’s Surgeon-in-Chief, wrote the first systematic report on frostbite injuries and their treatment [7]. In World War II, allied forces recorded 91,000 cases of frostbite, while German troops reported 46,000 cases [8].

Besides military personnel, other populations at risk of frostbite include psychiatric patients, homeless people, and people with a history of substance abuse [9]. A lack of adequate protection from the cold, either due to a lack of shelter or protective behaviours, is an important additional risk factor. A Canadian study reported that the most important predisposing factors for frostbite in Saskatchewans were alcohol consumption (46% of patients) and history of psychiatric illness (17% of patients) [10].

With the increase in people enjoying recreational activities in cold environments, and an increased accessibility to these environments for people with limited experience and/or inadequate preparation and equipment, the importance of frostbite has become more prevalent in such populations in recent years; additional risk factors include dehydration, high-altitude, and hypoxia, and pre-existing conditions such as peripheral vascular disease, diabetes mellitus, and Raynaud’s disease [11]. Of 24,079 climbers on Denali, Alaska, USA, between 1992 and 2011, 831 needed medical assistance, of which 171 were diagnosed with frostbite [12]. Out of 2941 patients treated in the medical facilities on the Everest base camp, Nepal, from 2003–2012, 77 were diagnosed with frostbite [13]. In the Teheran region, Iran, the incidence of frostbite in mountaineers was 366/1000 persons per year, with 83% being described as first grade injuries [14].

Many of the subjects that had suffered from frostbite complained about long-term sequelae [15,16,17,18,19,20,21,22]. Due to short periods of follow-up and the fact that most case reports in the literature do not address long-term sequelae, it is difficult to indicate an exact incidence of these conditions.

### 1.2. Pathophysiology

**Figure 1:**
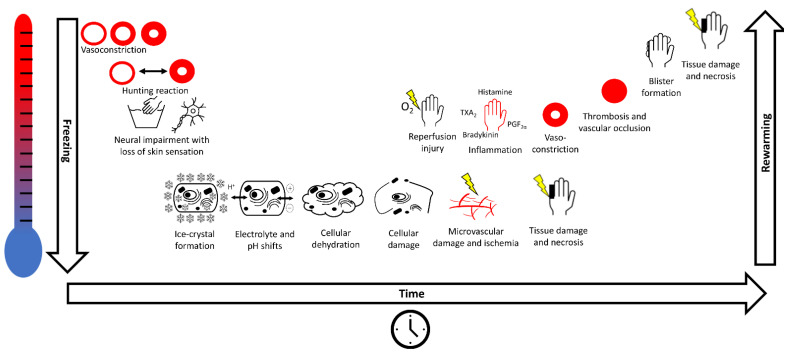
Pathophysiological cascade of frostbite. Cooling of the skin leads to vasoconstriction and to cycles of intermittent vasodilation and vasoconstriction, known as the “Hunting reaction”. Eventually neuronal impairment occurs with loss of sensation. Skin freezes with formation of extracellular ice-crystals that leads to electrolyte and pH shifts, cellular dehydration, and cell damage. Damage to the microvasculature results in ischaemia, tissue damage and necrosis, worsening over time. When skin is rewarmed, injury leads to inflammation, vasoconstriction, thrombosis, vascular occlusion, blister formation and eventually to tissue damage and necrosis.

Continued cold exposure of tissue leads to rapid vasoconstriction of the skin, which leads to further cooling and vasoconstriction of the subcutaneous tissue [23] (Figure 1). While cooling, human skin undergoes cycles of approximatively five to ten minutes, in which vasoconstriction is followed by vasodilatation, an adaptive response known as “Hunting reaction” [24]. Eventually, functional impairment of the neurons leads to loss of cutaneous sensation [3]. In the case of further cooling, vascular contents increase in viscosity and eventually the blood supply to the affected tissue gets shunted away to maintain core temperature, which in turn favours further tissue cooling.

While wind increases the speed of cooling (an effect known as “wind chill”), it does not affect the skin temperature [25]. Once freezing starts, extracellular and intracellular ice-crystal formation begins, which alters the osmotic gradient leading to electrolyte shifts, pH alterations, intracellular dehydration, and hyperosmolality, followed by harm to the cellular integrity of the affected subcutaneous tissue [26,27,28]. It has been proposed that a rapid-onset freezing injury (i.e., due to a higher temperature gradient and/or induced by a material with a high thermal conductivity) may initially lead to more intracellular ice-crystal formation while a slower-onset injury may lead initially to predominantly extracellular ice-crystal formation [29]. The damage to the microvascular endothelium of the affected tissue results in disruption of the microcirculation, leading to ischaemia and necrosis. After prolonged exposure to freezing temperature, ice-crystals increase in size, which directly damages cell membranes and extracellular structures. Of note, cartilage, especially epiphyseal cartilage, has been described to be particularly susceptible to frostbite injury [3].

Upon rewarming of frozen tissue, reperfusion injury with the generation of radical oxygen species (ROS) leads to an inflammatory response [30]. Data from burn models showed that prostaglandins, thromboxanes, bradykinin, and histamine are involved in oedema formation, endothelial injury, and subsequent arrest of dermal blood flow (Jackon’s zone of stasis) [31,32,33,34,35,36,37,38]. The presence of prostaglandin F_2α_ and thromboxane B_2_ (a stable metabolite of thromboxane A_2_), molecules that are known to be involved in vasoconstriction, platelet aggregation, and thrombosis, in both frostbite and burn injury blister fluid has been reported, indicating similar mechanisms of injury [39,40]. Subsequently, intravascular coagulation and oedema formation further sustain the reperfusion injury, potentially leading to showers of microthrombi damaging the microvasculature, and thrombus formation in larger vessels [3,39]. The outcome of frostbite injury depends on the degree of microvascular damage in any given tissue. Refreezing and subsequent rewarming, and thus multiple cycles of the pathophysiological cascade depicted in Figure 1, are related with a worse outcome.

Following acute pathology, frostbite long-term sequelae are associated with vasomotor dysfunction—in particular, vasospasm leading to circulatory disturbances, resulting in chronic pain and cold hypersensitivity. Also, cold-induced nerve damage is linked to neuropathic pain and ischemic neuritis. Furthermore, cold-induced arthritis is an important chronic condition that occurs in patients with a history of cold injury, which often only appears months or years later.

### 1.3. Classification

Frostbite classification is often performed using a scheme analogous to the classification of burn injuries, categorizing injuries in four different degrees [4]. In 2001, Cauchy et al. retrospectively reinterpreted the extent of the initial lesions of 70 patients with frostbite injuries, evaluated after rapid (1 h) rewarming of the tissue in warm (38 °C) water, and final amputation results. Based on these data and data obtained from isotopic bone scans, they proposed an alternative classification scheme based on the clinical appearance and anatomical extension of the frostbite lesion using the following grades: first grade: absence of initial lesion; second grade: initial lesion on distal phalanx; third grade: initial lesion on intermediate or proximal phalanx; 4th grade: initial carpal/tarsal lesion [41]. A comparison of the two classification systems is depicted in Table 1. Due to the still-sparse data available, there is currently no uniform classification of long-term sequelae of frostbite.

Comparison of the classification system proposed by Cauchy et al. [41] versus the traditional system that is based on the classification of burn injuries [4,42].

### 1.4. Management

Detailed descriptions of the broad spectrum of therapeutic options and recommendations for optimal frostbite treatment are published elsewhere (e.g., [1]). Briefly, frostbitten body parts should be protected from further cooling and mechanical damage. Since damage deteriorates during tissue freezing and rewarming, refreezing of tissue should be strictly avoided. In-field rewarming should only be considered if the risk of refreezing is negligible. Pain control should be achieved using nonsteroidal anti-inflammatory drugs (NSAID) and/or opioids. The need for hospital admission or outpatient treatment of frostbite patients must be assessed based on the severity of injury, comorbidities, medical history, and the need for in-hospital interventions (i.v. medication, radiology, surgery). Of note, during the first 24 h, the transfer of patients to a facility capable of administering thrombolysis, and, during the first 72 h, hospital admission for iloprost treatment, should be considered. Healing of frostbite can take from a few days up to several months and in some instances amputation of nonviable tissue is necessary [43,44]. For the management of frostbite long-term sequelae, Botulinum toxin has been proposed as a treatment for neuropathic and vasomotor difficulties [45]. Amitryptiline, gabapentinoids, duloxetine, and topical capsaicin or lidocaine, as well as epidural sympathectomy, is thought to be beneficial in chronic pain [46,47,48,49].

## 2. Methods

**Figure 2:**
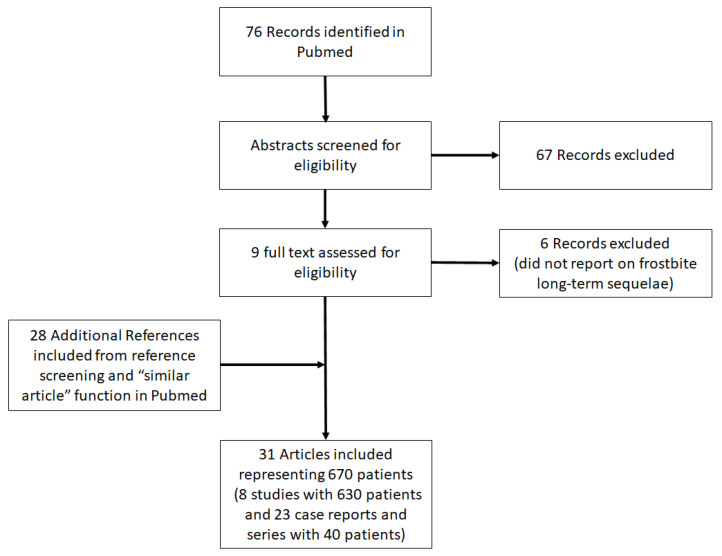
Preferred Reporting Items for Systematic Reviews and Meta-Analyses (PRISMA-ScR) flow chart for reference/literature selection [50].

In this review, cases regarding long-term sequelae from frostbite were identified and selected through a scoping literature review. This method was chosen because it was considered the most appropriate for identifying and mapping the available evidence on this topic. We followed to the Prisma-ScR checklist and scoping review guidelines (Figure 2) [50]. We did not establish or publish an a priori protocol for this study, and to our knowledge, none currently exists. For the purposes of this review, included cases conformed to all the following criteria: (1) cold injury from freezing temperatures, (2) ≥1 chronic ailment from cold injury, and (3) the possibility to retrieve a full-text version of the article online.

Literature published until 12 July 2021 was searched in PubMed using the following search terms: (“long term*”[All Fields] OR “long-term*”[All Fields] OR “chronic*”[All Fields]) AND (“frostbite*”[All Fields] OR “cold injur*”[All Fields] OR “cold-injur*”[All Fields]) AND (“aftereffect*”[All Fields] OR “aftermath*”[All Fields] OR “consequence*”[All Fields] OR “outcome*”[All Fields] OR “result*”[All Fields] OR “ramification*”[All Fields] OR “implication*”[All Fields] OR “repercussion*”[All Fields]). We did not limit the search to a specific article type, as long as the article was published in a peer-reviewed journal. We then used two additional searching techniques to increase the number of relevant articles. By entering the title of each article searched in the search field, we took advantage of the “similar articles” search option in PubMed. In addition, we searched the bibliography of retrieved articles for additional articles not found in the original searches. We only included articles in English. One author (IR) conducted the literature review and created the database. All reports that described any long-term effects of frostbite were considered, regardless of the level of detail. Articles not clearly distinguishing between frostbite and other pathologies (e.g., non-freezing cold injury) were excluded. Articles were categorized as either “study” or “case report/series”. We considered an article a study if data from various patients were synthesized into a tangible conclusion (e.g., the prevalence of a certain long-term sequelae). Data from studies are represented in a table depicting the population and follow-up timing, the grade or degree of frostbite, and the frequencies of patients suffering from long-term sequelae. Articles that described the signs or symptoms and possible treatments and outcomes of individual patients we considered case reports/series. Data from case reports/series are represented in a supplementary table including the patient’s age, grade/degree of frostbite, sex, location of the injury, time since the initial frostbite injury, signs, and symptoms, as well as treatment and outcome.

## 3. Results

The database search identified a total of 76 references (Figure 2). A total of 67 references were excluded after reading the title and abstract and 9 were retrieved for further evaluation. Of these, 6 studies were excluded, because they did not report on long-term sequelae after frostbite injury. Following the selection process and additional reference screening, 8 studies plus 23 other case reports were included in the scoping review. Table 2 shows the results of 8 studies regarding long-term sequelae of frostbite.

Among 493 patients from five studies in which long-term sequalae could be clearly attributed to individuals, 341 were affected (69%) [15,16,17,18,21]. Three of seven studies reported long-term sequelae in 56 civil patients and four studies in 574 military persons. The incidence and type of long-term sequelae (i.e., neuropathy-related signs and symptoms) were similar between different studies and in the two populations examined (civil vs. military), that included mostly patients with 1–2 frostbite grade/degree. Two thirds of the members of the Norwegian armed forces that sustained frostbite reported suffering from sequelae until two years after the initial injury, with 21% saying that their ability to work and their leisure activities were negatively affected [15]. In a Swedish study, most of the patients were still suffering from long-term sequelae four years after the initial injury. 73% of patients that reported pain and discomfort in the affected tissue four months after frostbite injury had pathological findings when undergoing quantitative sensory testing (thermal and vibrotactile perception threshold) [16]. Patients treated for frostbite in the urban area of Helsinki reported suffering from chronic pain, with 15% even suffering from intolerable daily chronic pain two to nine years after the frostbite injury [17]. Another Finnish paper from Lapland reported that 63% of 30 patients treated for frostbite in the previous 4–11 years suffered from long-term sequelae [18]. 38 of 40 Norwegian soldiers who suffered from frostbite of first to third degree during their service reported chronic ailments [19,20]. The US army reported that 65% of inpatients treated for frostbite suffered symptoms such as cold sensitivity, paraesthesia, pain, and hyperaesthesia six months after the initial injury [21]. During the Korean war, the vast majority of US soldiers evacuated due to frostbite injury suffered from either hyperhidrosis, chronic pain, cold hypersensitivity, paraesthesia, and/or arthritis four years after injury [22].

In addition, our literature search retrieved 23 case reports and series, including 40 patients suffering from frostbite long-term sequelae (Table S1) [45,51,52,53,54,55,56,57,58,59,60,61,62,63,64,65,66,67,68,69,70,71,72]. Most of the case reports describe frostbite-related arthritis that clinically and radiologically resembles osteoarthritis. Due to the insidious symptom progression in patients suffering from frostbite arthritis, diagnosis is often delayed—sometimes for decades. Frostbite during childhood was associated with epiphyseal plate damage leading to deformities of affected fingers. Of note, ten cases of squamous cell carcinoma on the heel that developed 25 to 35 years after initial frostbite injury have been reported in Greek World War II veterans [59].

## 4. Discussion

The main findings of the present scoping review are that data about long-term sequelae from frostbite are scarce and, in many publications, patients were not followed-up for the long term. Among 493 patients from five studies in which long-term sequalae could be clearly attributed to individual patients, 341 were affected (69%). Long-term sequelae included neuropathy-related signs and symptoms like chronic dysesthesia, chronic pain, and hyperhidrosis, often leading to functional impairment. Such long-term sequelae were common even with frostbites of first and second grade in healthy individuals. It must be noted that the definition of long-term sequalae is subject to debate since spontaneous recovery from acute injury can take several months [73]. We included studies and cases that reported sequalae that appeared after conclusion of treatment of the acute injury, regardless of the time after injury. This resulted in data from 4 months to 50 years after injury.

**Figure 3:**
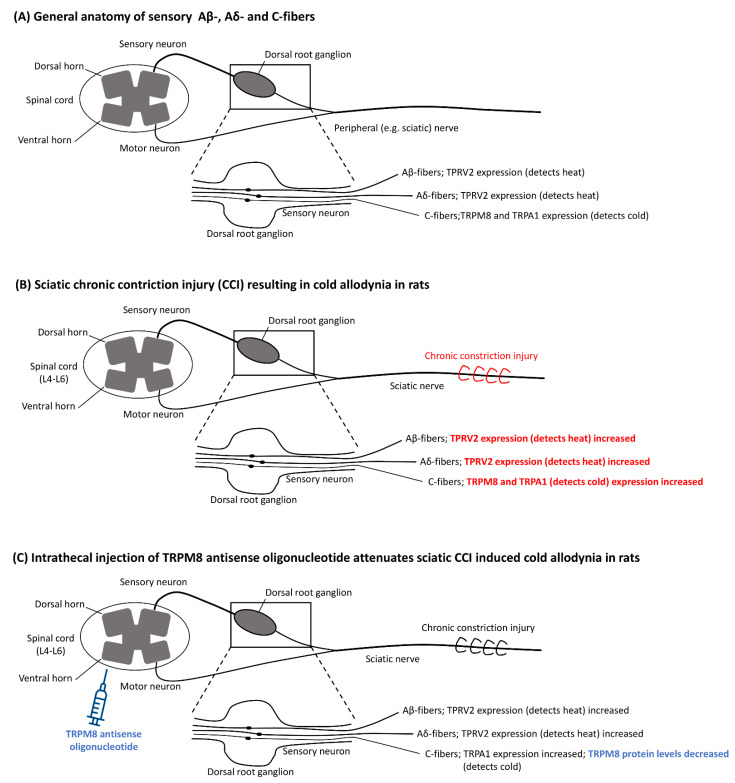
The role of the transient receptor potential channel (TRP) family in cold allodynia. (**A**) Sensory Aβ- and Aδ-fibres express TRPV2 activated by heat stimulus > 52 °C. C-fibres express TRPA1 activated by cold (5–18 °C) and TRPM8 activated by cold (<20 °C). (**B**) During sciatic CCI, cold allodynia was associated with expression of TRPV2, TRPA1, and TRPM8 in the ipsilateral dorsal root ganglia at L4–L6. (**C**) Intrathecal injection of TRPM8 antisense oligonucleotide attenuated cold allodynia and decreased TRPM8 protein levels in the ipsilateral dorsal root ganglia of L4–L6. TRPV2: Transient receptor potential cation channel, subfamily V, member 2; TRPA1: Transient receptor potential cation channel, subfamily A, member 1; TRPM8: Transient receptor potential cation channel, subfamily M, member 8; CCI: Chronic Constriction Injury; L: Lumbar vertebra.

After frostbite lesions, vasomotor disturbances—notably vasospasm—leading to impaired circulation are associated with chronic pain and cold hypersensitivity [74]. Data from an animal model indicates that frostbite injury leads to a decrease in endothelial nitric oxide production, possibly predisposing affected tissue to vasoconstriction [75]. Furthermore, it has been proposed that higher sympathetic drive during cooling of frostbite-injured tissue may contribute to vasoconstriction and cold sensitivity [5] Cold sensitization, also termed allodynia, is caused by inflammation that lowers the threshold for pain perception, allowing innocuous stimuli to produce pain in the sensitised tissue. Chronic pain includes cold allodynia (excessive reaction to all stimuli, including those not normally evoking pain, that radiates to adjacent areas and persists beyond the stimulus), hypoesthesia (partial loss of all forms of sensation), paraesthesia (spontaneous positive, prickling sensation), hyperaesthesia (increased cutaneous sensitivity to various stimuli) and hyperhidrosis (due to overactivity of sudomotor nerve fibres), all of which have been ascribed to cold-induced nerve damage [18]. All these symptoms were variably reported in the eight studies reviewed [15,16,17,18,19,20,21,22]. In patients with freezing cold injuries, a loss of small and large neuronal fibre function has been evidenced, suggesting that pain has its origin in neuropathy [76].Indeed, Carlsson et al. documented in a series of 40 soldiers with such long-term sequelae a decrease in nerve conduction velocity (both motor and sensory), and an increased motor distal delay consistent with neuropathy [16]. This damage has been suggested to be due to direct cold nerve injury (e.g., from ice-crystal formation) or to retrograde axonal degeneration after peripheral axonotmesis [77]. Furthermore, patients with long-term sequelae have shown a heavily delayed or abolished Hunting reaction in the affected tissue [78]. At the same time, they were prone to vasospasm and had a pronounced decrease in skin temperature upon exposure to cold air [18]. In a rat model, partial freezing of the sciatic nerve has induced transient thermal hyperalgesia, while complete freezing led to anaesthesia [79]. Transient receptor potential cation channel, subfamily M, member 8 (TRPM8) is an excitatory ion channel expressed on Aδ and C-fibres, which is activated at temperatures < 20 °C [80,81,82,83] (Figure 3).

It has been shown that menthol topical application induced cold allodynia via activation and sensitisation of TRPM8 (being both a menthol and cold-sensitive ion channel) [81,82]. It is thought to be the main detector of environmental cold in mice and is part of the transient receptor potential channel (TRP) family, which also includes TRPA1 and TRPV2 [84,85,86,87]. TRPA1 is expressed on C-fibres and is also activated by noxious cold (5–18 °C) [88]. In contrast, TRPV2 is found on larger and myelinated sensory Aβ- and Aδ-fibres and is activated upon heat (>52 °C) [89]. Furthermore, TRPA1 and TRPM8 act as mediators for vasoconstriction upon tissue cooling [90]. The sciatic nerve chronic constriction injury (CCI) rat model is widely used to study peripheral nerve injury [91,92]. Frederick at al. reported that TRPA1, TRPM8, and TRPV2 expression was upregulated in the ipsilateral dorsal root ganglia of L4–L6 after sciatic nerve CCI [93]. These findings suggest that freezing-induced peripheral nerve damage and subsequent upregulation of the beforementioned receptors play a role in frostbite-induced temperature hypersensitivity. Accordingly, Su et al. found elevated TRPM8 protein levels in the ipsilateral L5 ganglion and reported that rats suffered from cold allodynia after sciatic nerve CCI [94]. Intrathecal administration of TRPM8 antisense oligonucleotides attenuated cold allodynia and decreased TRPM8 protein levels. Calvo et al. also showed that TRPM8 antagonists are effective against cold allodynia in rats [95]. However, Namer et al. reported that topical administration of a 20% cinnamaldehyde (TRPA1 agonist) or a 40% menthol (TRPM8 agonist) solution on patients suffering from frostbite-related cold allodynia did not show any sensitisation compared to healthy controls, suggesting that cold allodynia in these patients is not related to the overexpression of these receptors [96]. Thus, the exact role of the TPR family in thermal hyperalgesia secondary to frostbite is still under debate and needs further investigation.

In addition, frostbite arthritis is another important sequelae found in patients with previous frostbite injury. Clinically resembling regular osteoarthritis, it usually appears month or years after the initial frostbite injury. The underlying bone and cartilage damage are thought to be mostly a consequence of circulatory insufficiency during frostbite injury, although direct cold damage may also be involved [54]. In children, the higher specific surface increases the risk of frostbite injury and severity and can lead to epiphyseal cartilage damage and subsequent abnormal growth of the affected bones [54,56,58,60,63,70,97].

In many cases, the management of chronic neuropathic pain after frostbite is challenging; duloxetine, amitriptyline, and gabapentinoids such gabapentin or pregabalin might be beneficial. Also, topical administration of capsaicin or lidocaine are thought to alleviate painful frostbite sequalae [46,47,49]. Furthermore, epidural sympathetic blockade has been described as an effective temporary and non-destructive treatment [48]. Long-lasting sympathectomies can be achieved chemically (e.g., alcohol or phenol injection) and surgically (e.g., open removal or electrocoagulation of sympathetic chain, or minimally invasive stereotactic thermal or laser interruption) [98]. Also, surgical sympathectomy [48,74], vasodilators, and β-blockers have been reported to ameliorate symptoms [99]. Several studies reported positive effects of Botulinum toxin type A (BTX-A) injections in patients with Raynaud’s phenomenon, that is characterised by inappropriate vasoconstriction in the distal extremities upon cold stimulation [100]. BTX-A blocks acetylcholine-mediated signalling to the vasculature smooth muscle, preventing vasoconstriction. In addition, it has been shown that BTX-A impedes sympathetic vasoconstriction by blocking noradrenaline vesicle exocytosis at the vascular smooth muscle neuromuscular junction in a guinea-pig model [101]. Moreover, it has been shown that BTX-A decreases the activity of C-fibre nociceptors through inhibition of the release of local nociceptive neuropeptides (e.g., calcitonin gene-related peptide, substance P, glutamate). All these mechanisms are thought to act together, reducing cold-induced vasoconstriction and pain [101,102]. There is evidence that intradermal, subcutaneous, or perineural injection of BTX-A is useful in the treatment of chronic neuropathic pain [103,104]. Injection around the neurovascular bundles in the palm of frostbite-injured fingers in a patient suffering from cold hypersensitivity and sensory-motor disturbances secondary to frostbite successfully mitigated most of these symptoms in a case report [45]. In the same case, infrared thermography measurement showed that BTX-A treatment did improve skin rewarming after a mild cold provocation test (which implies an improvement of perfusion), and quantitative sensory testing showed an improvement in sensory function. Frostbite arthritis patients may benefit from non-pharmacological interventions such as physiotherapy [51]. There is one report about clodronic acid having some therapeutic benefits, while non-steroidal anti-inflammatory drugs are thought to be of little benefit [53]. Due to the complexity of refractory pain after frostbite, a chronic pain specialist should be included in a multidisciplinary approach [46,47,49].

## 5. Limitations

The scarcity of data on long-term sequelae of frostbite is the major limitation of this review. 

## 6. Conclusions

In the 670 frostbite patients who were followed up, many suffered from long-term sequelae. These include neuropathy-related signs and symptoms (chronic dysesthesia, chronic pain, and hyperhidrosis), as well as arthritis—even after lower-grade frostbite in healthy individuals. Botulinum toxin is a promising therapeutic option for neuropathic and vasomotor difficulties that needs further research. Epidural sympathectomy is another beneficial intervention for temporary symptom relief. More in-depth research into the pathogenesis of cold allodynia and the role of the TRP family may lead to new specific treatments of neuropathic and vasomotor difficulties. The treatment of frostbite arthritis with clodronic acid has been described as useful in one case report and needs further investigation. 

A better understanding of the exact injury mechanism could help to determine the extent to which it might be possible to intervene pharmacologically in a potentially chronic inflammatory process.

## Figures and Tables

**Table 1:** Classification of frostbite.

Classification Proposed by Cauchy et al.		Classification Analogous to Burn Injury		
	**Extent of Initial Lesion** **Immediately after Rewarming**		Lesion Characteristics *	**Time to Onset** **After Rewarming**
first Grade	Absence of initial lesion	first Degree	Partial skin freezing with: Erythema, oedema Skin desquamation	2–3 h 5–10 days
second Grade	Initial lesion on distal phalanx	second Degree	Full thickness skin freezing with: Clear blister formation Intensive pain	12–24 h 3–10 days
third Grade	Initial lesion on intermediate or proximal phalanx	third Degree	Subcutaneous freezing with: Haemorrhagic blister formation Skin necrosis	12–24 h 5 days–5 weeks
fourth Grade	Initial carpal/tarsal lesion	fourth Degree	Freezing deeper than the subcutis with: Cyanotic and insensitive tissue Tissue mummification	Immediately up to 3 months

* Lesion characteristics for a specific degree include those described plus those of all the lower degrees.

**Table 2:** Studies reporting frostbite long-term sequelae.

Article	Population and Follow Up Timing	Frostbite Grade/Degree	Long-Term Sequelae
Norheim et al., 2018 [15]	Self-reported data of 397 Norwegian soldiers in 2017 having suffered frostbite from 2010–2014	1–2	70 % with long-term sequelae 21% unable to work and undertake usual leisure activities
Carlsson et al., 2014 [16]	Self-reported data of 12 patients; 4 patients with hand frostbite, 6 patients with feet frostbite, and 2 patients with hand and feet frostbite; hand frostbite was followed-up after 4 month and 4 years, foot frostbite only after 4 years	1–2	4 months after frostbite of the hands (*n* = 6): 100% with discomfort when exposed to cold 67% with cold sensation 67% with white fingers/toes 4 years after frostbite of the hands (*n* = 6): 100% with discomfort when exposed to cold 83% with cold sensation 17% with white fingers/toes 4 years after frostbite of the feet (*n* = 8): 89% with discomfort when exposed to cold 100% with cold sensation 100% with white fingers/toes
Koljonen et al., 2004 [17]	Self-reported data form 14 patients with frostbite during the previous 7 years	Not specified	15% with daily, intolerable pain 50% chronic pain 50% with limitations in their social life 36% with poor emotional well being
Ervasti et al., 2000 [18]	Clinical examination of 30 patients with frostbite 4–11 years earlier	2	63% with sequelae of any kind 66% with increased tendency for vasospasm 53% with hypersensitivity to cold 40% with numbness of fingers 33% with declined sensitivity to touch 13% with lowered working ability
Arvesen et al., 1996 * [19]	Clinical examination of 40 Norwegian soldiers with frostbite in the previous 21–78 months; 16 with involvement of the hands and 24 with involvement of the feet	1–3	38% with disturbed sense of cold 38% with disturbed sense of heat 33% skin and nail dystrophia 20% with hyperhidrosis 18% with reduced light-touch perception 18% with reduced pain perceptions 10% with reduced blunt-touch perception 8% with pain on deep pressure5% with paraesthesia 3% with reduced muscle power
Rosen et al., 1991 * [20]	Self-reported data of 40 Norwegian soldiers with frostbite at least 2 years prior; 18 with involvement of the hands and 28 with involvement of the feet	1–3	Hands: 100% with cold hypersensitivity 50% with paraesthesia 61% with hypaesthesia 56% skin and nail dystrophia 44% with pain 6% with hyperaesthesia 6% with hyperhidrosis 6% with arthralgia Feet: 93% with cold hypersensitivity 64% skin and nail dystrophia 54% with pain 46% with paraesthesia 54% with hypaesthesia 14% with hyperhidrosis 11% with hyperaesthesia 7% pain when walking 4% with arthralgia
Taylor et al., 1989 [21]	40 US soldiers examined 6 months after frostbite	1–4	65% with neurovascular sequelae (cold sensitivity, paraesthesia, pain, and hyperaesthesia) 8% had to be reassigned to new functions due to symptom severity
Blair et al., 1957 [22]	Self-reported data of 97 US soldiers with frostbite in previous 4 years; 50 were examined clinically	2–4	Self-reported sequelae in winter 71% with numbness 70% with pain 69% with cold feet 58% with abnormal colour 53% with hyperhidrosis 40% with pathology in joints Self-reported sequelae in summer 31% with numbness 45% with pain 24% with cold feet 31% with abnormal colour 78% with hyperhidrosis 25% with pathology in joints Sequelae detected on physical examination 58% with abnormal nails 48% with abnormal colour 42% with hyperhidrosis 28% with joint stiffness

* Studies about the same subjects.

## Supplementary Materials

The following are available online at https://www.mdpi.com/article/10.3390/ijerph18189655/s1, Table S1: Case reports and case series about frostbite long-term sequelae.
