# Supplementary material for: Long-Term Sequelae of Frostbite—A Scoping Review
Source: Int J Environ Res Public Health. 2021 Sep 14;18(18):9655. doi: 10.3390/ijerph18189655 (PMC8465633; doi:10.3390/ijerph18189655)
Supplement: Supplementary file 1 [file ijerph-18-09655-s001.zip › ijerph-1344253-supplementary.pdf]

Table S1: Case reports and case series about frostbite long-term sequelae. Grades of frostbite are not specified except in Irsay et al., 2019 [51] as grade 3

**Article**	**Age**	**Sex**	**Location of injury**	**Years since frostbite**	**Signs and symptoms**	**Treatment and Outcome**
Irsay et al., 2019 [51]	35	Male	Both hands	17	Functional impairment, hyperalgia, histophatological alterations of cartilage	Not specified
Norheim et al., 2017 [45]	24	Male	Both hands	2	Cold intolerance, loss of sensation, pain	Not specified
Wang et al., 2016 [52]	35	Male	Both hands	17	Stiffness and weakness in hands, enlargement of the interphalangeal joints, shortening of digits	Not specified
Kahn et al., 2005 [53]	46	Female	Both hands	22	Deformations and pain in the interphalangeal joints	Clodronic acid 800 mg 2x / d leading to clinical improvement
Pettit et al., 1998 [54]	29	Male	Both hands	Injury during childhood	Pain, swelling decreased range of motion in the interphalangeal joints	Paracetamol and naproxen led to clinical improvement
Turner et al., 1998 [55]	36	Male	Right hand	14	Swelling and reduced flexion in the interphalangeal joints	Not specified
Crouch et al., 1990 [56]	14	Male	Both hands and feet	13	Limited motion of hands and feet, deformitiy of fingers and toes	Not specified
Leung et al., 1985 [57]	9	Male	Both hands	5	Deformity and ulnar deviation of fingers	Not specified
Nakazato et al., 1985 [58]	4	Female	Both hands	2	Enlarged interphalangeal joints, radial deviation, and flexion deformity of fingers	Not specified
Rossis et al., 1982 [59]	52 - 62	10 Men	Heel	29-38	Squamous cell carcinoma	Surgical excision
Carrera et al., 1981 [60]	16	Female	Both hands	14	Finger deformities and stiffness of the interphalangeal joints	Not specified
McKendry et al., 1981 [61]	29	Male	Both hands	15	Swelling and stiffenss of the proximal interphalangeal joints, flexion deformity of the distal phalanges, cold intolerance	Not specified

Solomon et al., 1980 [62]	22	Female	Both hands	20	Pain and swelling in interphalangeal joints	Not specified
Carrera et al., 1979 [63]	14	Male	Both hands	1	Swollen interphalangeal joints with morning stiffness	Not specified
Carrera et al., 1979 [63]	25	Male	Left hand	5	Flexion deformities and crepitation in the distal interphalangel joints	Not specified
Ellis et al. 1969 [64]	44	Female	Left hand	27	Swelling of the distal interphalangeal joints	Not specified
Selke et al., 1969 [65]	9	Female	Both hands	6	Pain	Not specified
Lindholm et al., 1968 [66]	6	Male	Both hands	3	Radial deviation of fingers, cold intolerance	Not specified
Lindholm et al., 1968 [66]	12	Male	Both hands	8	Deformitiy of fingers	Not specified
Lindholm et al., 1968 [66]	21	Female	Both hands	18	Deformitty of fingers	Not specified
Wenzl et al., 1967 [67]	10	Male	Both hands	1	Swelling of interphalangel joints with flexion deformity	Not specified
Bigelow et al., 1963 [68]	58	Male	Both hands	50	Shortening and deformity of fingers	Not specified
Bigelow et al., 1963 [68]	51	Female	Right hand	48	Deformity and extension weakness of fingers, cold intolerance	Not specified
Bigelow et al., 1963 [68]	12	Female	Both hands	12	Finger deformities, imparied flexion or extension in different joints	Not specified
Bigelow et al., 1963 [68]	11	Male	Both hands	9	Deformitiy of fingers, cold intolerance	Not specified
Bigelow et al., 1963 [68]	37	Female	Left hand	34	Deformity of fingers, hyperalgia	Not specified
Bigelow et al., 1963 [68]	13	Female	Right hand	12	Deformity of hand, muscular weakness	Not specified
Flortkiewicz et al., 1961 [69]	5	Male	Both hands	3	Deformity of fingers	Not specified
Dreyfuss et al., 1955 [70]	7	Female	Both hands	5	Deformity of fingers	Not specified
Thelander 1950 [71]	9	Male	Right hand	2	Pain and swelling in the interphalangeal joints	Not specified

Bennet et al., 1935 [72]	8	Female	Left hand	7 months	Swelling and limitaion of motion of the proximal interphalangelal joints	Temporary splinting of fingers did not improve outcome
